# Functional validation of a *white pupae* minimal gene construct in *Ceratitis capitata* (Diptera: Tephritidae)

**DOI:** 10.1111/1744-7917.70058

**Published:** 2025-05-12

**Authors:** Lucas Henrique Figueiredo Prates, Roswitha A. Aumann, Inga Sievers, Tanja Rehling, Marc F. Schetelig

**Affiliations:** ^1^ Department of Insect Biotechnology in Plant Protection Justus Liebig University Giessen Giessen Germany

**Keywords:** gene editing, genetic rescue, Mediterranean fruit fly, *piggyBac*, selectable marker, SIT

## Abstract

Genetic sexing strains (GSS) are important tools for the sterile insect technique (SIT), an environmentally friendly and species‐specific insect pest control method. GSS feature sex‐specific phenotypes, enabling sex sorting in mass‐rearing facilities and male‐only releases, which significantly improve the cost‐effectiveness and efficiency of SIT programs. In classical GSS, sex linkage of marker gene(s), such as *white pupae* (*wp*), is achieved through an irradiation‐induced translocation between the marker‐carrying autosome and the Y chromosome. However, this approach may render GSS males semisterile. The recently proposed neo‐classical GSS concept suggests using genome editing to achieve sex linkage by directly inserting the wild‐type marker allele onto the Y chromosome, potentially yielding GSS males with higher fertility. In this study, we examined the *Ceratitis capitata wp* gene as a genetic marker for the neo‐classical GSS concept and developed a minimal, intronless version of this gene, termed mini‐*wp*. We demonstrate that a single copy of mini‐*wp* is sufficient to restore the wild‐type brown puparium phenotype and is functional when integrated at various positions within the *C. capitata* genome, including the X chromosome. Due to its smaller size (4689 bp, including 2000 bp of putative promoter region) relative to the full wild‐type *wp* allele (20868 bp), mini‐*wp* may facilitate its precise insertion into the Y chromosome, representing an important step toward realizing neo‐classical GSS. Furthermore, the methodology developed for designing and testing mini‐*wp* in medfly may be adapted to other Tephritid species with an identified *wp* gene.

## Graphical abstract

An intronless version of the *white pupae* (*wp*) gene was engineered to restore the wild type brown puparium color in white pupae phenotype mutants of the Mediterranean fruit fly, *Ceratitis capitata*. Functionality of the minimal gene version (mini*‐wp*) was verified *in vivo*, as one copy of mini‐*wp* successfully restored the wild type phenotype in homozygous *wp^‐^* mutant individuals. The herein presented molecular techniques and work‐pipeline could be used as a toolbox to generate sexing systems in other insect pest species with a white pupae phenotype.

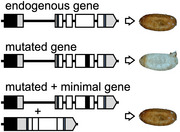

## Introduction

Genetic sexing strains (GSS) are a proven valuable tool for sex‐sorting in insect mass‐rearing facilities and a key element for cost‐effective application of the sterile insect technique (SIT) for pest control (Franz *et al.*, 2021; Klassen *et al.*, 2021; Mumford, 2021). SIT programs are based on mass rearing of the target insect, followed by its sterilization and release into the infested area. Sterile males mate with wild females, leading to no offspring, thus gradually reducing the insect population in the treated area (Knipling, 1955). A GSS classically requires two components: a selectable mutated marker that allows sex separation or elimination of females at the earliest possible stage, and linkage of the wild‐type (WT) allele of this marker to the male sex. A well‐known and successful example of a GSS is the VIENNA‐8, developed in *Ceratitis capitata* (Wiedemann) (Diptera: Tephritidae), commonly known as the Mediterranean fruit fly, or medfly (Franz *et al.*, 1994; Augustinos *et al.*, 2017; Franz *et al.*, 2021). This GSS carries two selectable markers, *white pupae* (*wp^−^*) and *temperature‐sensitive lethal* (*tsl^−^*), originally located on an autosome (chromosome 5). An irradiation‐induced translocation has moved the WT alleles of both markers onto the Y chromosome, facilitating sex separation; female flies, homozygous for the mutated alleles, emerge from white puparia and are sensitive to high temperatures. In contrast, heterozygous male flies emerge from brown puparia and are not affected by these high temperatures. Typically, during operations in mass rearing facilities, female embryos do not survive a treatment at 34 °C for 24 h, while males are unaffected (Franz *et al.*, 2021).

However, the establishment of GSS strains has been a resource‐intensive process, primarily governed by stochastic factors. For example, the *wp^−^* phenotype in Tephritidae, arising from natural mutations, was so far only isolated in the medfly (Rössler, 1979), the melon fly (*Zeugodacus cucurbitae*) (McInnis *et al.*, 2004), and the oriental fruit fly (*Bactrocera dorsalis*) (McCombs & Saul, 1992, 1995). Furthermore, GSS based on a translocation of the autosomal WT alleles onto the Y chromosome may, depending on the Y chromosome translocation breakpoint and the resulting segregation behavior during male meiosis, be semisterile and genetically unstable, that is, autosomal recombination might occur during mass rearing, potentially compromising the GSS (Franz *et al.*, 2021; Cáceres *et al.*, 2023). Therefore, the development of general tools for faster development of GSS remains necessary for boosting and expanding SIT to other insect species (Bourtzis & Vreysen, 2021).

Although the *wp^−^* phenotype has been used in GSS for decades, the genetic basis for this phenotype in Tephritids has only recently been revealed (Ward *et al.*, 2021), enabling the creation of multiple CRISPR/Cas‐based lines with the *wp^−^* phenotype in the medfly, the Queensland fruit fly, *B. tryoni*, and the melon fly (Ward *et al.*, 2021; Paulo *et al.*, 2022). The identification of this and additional suitable marker genes (Robinson, 2002; Chen *et al.*, 2022; Sollazzo *et al.*, 2024; Paulo *et al.*, 2025), combined with ongoing efforts to generate high‐quality complete reference genomes of arthropods of agricultural and public health interest (Matthews *et al.*, 2018; Palatini *et al.*, 2020; Childers *et al.*, 2021; Fisher *et al.*, 2022; Wang *et al.*, 2023; Zhang *et al.*, 2023), and recent advancements in genetic editing in insects using modern molecular techniques (Aumann *et al.*, 2018; Buchman & Akbari, 2019; Meccariello *et al.*, 2019; Aumann *et al.*, 2020; Gamez *et al.*, 2021; Häcker *et al.*, 2021; Yan *et al.*, 2023), enables the concept of the “neo‐classical genetic approach” to generate GSS. In this approach, sex linkage could be achieved by inserting the WT allele of selectable marker(s) into the Y chromosome or near the male determining factor in a strain with mutated markers (Nguyen *et al.*, 2021; Yan *et al.*, 2023; Yan *et al.*, 2024). Because the CRISPR/Cas homology‐directed repair (HDR)‐mediated insertion efficiency is sensitive to the size of the insert and may improve with smaller cargos (Li *et al.*, 2014; Paix *et al.*, 2017), using engineered minimal gene constructs instead of the full endogenous allele could increase editing success. This might be particularly relevant for the development of neo‐classical GSS, where targeting a gene‐poor and highly repetitive Y‐chromosome is required (Charlesworth & Charlesworth, 2000; Bachtrog, 2013; Choo *et al.*, 2019).

Here, we examine the *wp* gene as a genetic marker for the neo‐classical GSS concept in *C. capitata*, and report the successful restoration of the WT brown pupae phenotype in the medfly by *piggyBac* integration of an intronless version of the *white pupae* gene into a *wp* ^−^ strain.

## Materials and methods

All primers used in this study were designed with Geneious Prime (version 2021.2.2; Kearse *et al.*, 2012) and are listed in Table S1.

### Insect strains and rearing

*Ceratitis capitata* (Wiedemann) strains wild‐type Egypt‐II (EgII), *white eye* (*we* ^−^), and *white pupae* (*wp* ^−^) were obtained from the Insect Pest Control Laboratory, Joint FAO/IAEA Centre of Nuclear Techniques in Food and Agriculture (IPCL/IAEA, Seibersdorf, Austria). The CRISPR‐modified *wp^−^* strain (*wp* ^−(CRISPR)^) was previously established in the laboratory (strain *Cc_D*) (Ward *et al.*, 2021). The strain double homozygous for the *white eye* and *white pupae* natural mutations (*we* ^−^ *wp* ^−^/*we* ^−^ *wp* ^−^) was produced by inbreeding the naturally mutated *white eye* and the naturally mutated *white pupae* strains. All insect strains were reared under standard laboratory conditions at 25 ± 1 °C, 48% relative humidity (RH), and 14 h : 10 h light/dark cycle. Larvae were reared on carrot‐based larval food prepared with 1.4 kg of cooked frozen carrots, 16 g of sodium benzoate (VWR International GmbH, Darmstadt, Germany), 500 g of carrot powder (Van Drunen Farms, Momence, IL, USA), 168 g of yeast hydrolysate enzymatic (MP Biomedicals, Solon, OH, USA), 20 mL of hydrochloric acid solution 25% (v/v) (Carl Roth GmbH + Co. KG, Karlsruhe, Germany), blended with approximately 2 L of distilled water to adjust to a smooth consistency. Adults were fed *ad libitum* with a mixture of sugar and yeast hydrolysate enzymatic (MP Biomedicals) (3 : 1, v : v), and water.

### Rapid amplification of cDNA ends

Total RNA was extracted from single pre‐pupa of EgII strain using Monarch total RNA Miniprep kit (New England Biolabs Inc., Ipswich, MA, USA) following the manufacturer's instructions. 5 *µ*g of DNA‐free total RNA was used to isolate mRNA using the NEBNext Poly(A) mRNA Magnetic Isolation Module (NEB #E7490, New England Biolabs Inc., Ipswich, MA, USA). Both 5'‐ and 3'‐rapid amplification of cDNA ends (RACE) was performed using SMARTer^®^ RACE 5'/3' Kit (Takara Bio USA, Inc., Mountain View, CA, USA) following the manufacturer's instructions with primers P2125 and P2126. Cycler conditions were as follows: 5 cycles 94 °C for 30 s, 72 °C for 3 min, 5 cycles 94 °C for 30 s, 70 °C for 30 s, 72 °C for 3 min, 25 cycles 94 °C for 30 s, 65 °C for 30 s, 72 °C for 3 min. PCR products were analyzed via gel electrophoresis, extracted using Zymoclean Gel DNA Recovery Kit (Zymo Research Europe GmbH, Freiburg, Germany), cloned into the linearized pRACE vector (Takara Bio USA, Inc.), and transformed into XL1‐Blue MR Supercompetent cells (*E. coli* Δ(mcrA)183 Δ(mcrCB‐hsdSMR‐mrr)173 endA1 supE44 thi‐1 recA1 gyrA96 relA1 lac [F proAB lacIqZΔM15 Tn10 (Tetr)]; Agilent Technologies, Santa Clara, CA, USA). Individual colonies were grown in LB medium containing 100 ng/*µ*L ampicillin (Carl Roth GmbH + Co. KG, Karlsruhe, Germany) and plasmids were extracted using NucleoSpin Plasmid Mini kit (Macherey‐Nagel GmbH & Co. KG, Düren, Germany). Finally, plasmids were pre‐selected by restriction digestion with EcoRI‐HF and HindIII‐HF (New England Biolabs Inc., Ipswich, MA, USA) and Sanger‐sequenced (Macrogen). Sequencing results were aligned to the medfly reference genome version 2.1 (GCF_000347755.3) (Papanicolaou *et al.*, 2016).

### *White pupae* minimal gene construct

The construction of the minimal gene construct for the *white pupae* gene (termed mini‐*wp*) involved amplifying a 2 kb region upstream of the 5' UTR, which is assumed to contain the promoter, along with part of the 5' UTR itself (521 bp). This was done using primers P2237 and P2238 on genomic DNA (gDNA) extracted from a virgin EgII female adult. The coding sequence, together with part of the 5' UTR and the 3' UTR (2168 bp in total), were amplified using 3 *µ*L of 1: 5 diluted cDNA from a single pre‐pupae of the strain EgII, prepared as described above for RACE PCR, using primers P2239 and P2240 spanning from the annotated 5' UTR to the end of the 3' UTR (Fig. S1). Phusion Flash High‐Fidelity PCR Mastermix was used for both amplifications and cycler conditions were as follows: 98 °C for 10 s, 35 cycles 98 °C for 1 s, 50 °C for 5 s, 72 °C for 1 min, 72 °C for 3 min. PCR products were analyzed via gel electrophoresis, extracted using Zymoclean Gel DNA Recovery Kit (Zymo Research Europe GmbH, Freiburg, Germany), and assembled into the SacII/XhoI‐digested *piggyBac* transformation vector *AH465* (*pXLBacII_IE1hr5‐DsRed.T3‐SV40*) (Li & Handler, 2017) using the Gibson Assembly Cloning Kit (New England Biolabs Inc., Ipswich, MA, USA). The resulting plasmid *M6620* (*pXLBacII_mini‐wp_IE1hr5‐DsRed.T3‐SV40*) was used to transform XL1‐Blue MR Supercompetent cells (Agilent Technologies, Santa Clara, CA, USA). Correct assembly and sequence of the mini‐*wp* insert (in total 4689 bp, including 2000 bp of putative promoter region) was verified by restriction digestion with NgoMIV and AfiII and Sanger sequencing. Finally, endotoxin‐free plasmid DNA was prepared using the purification kit NucleoBond^®^ Xtra Maxi EF (Macherey‐Nagel GmbH & Co. KG, Düren, Germany), following manufacturer's instructions.

### Germline transformation

Germline transformation was achieved through microinjection into embryos of the *C. capitata wp* ^−(CRISPR)^ strain (Ward *et al.*, 2021), using a mixture of KCl (5 mmol/L) and NaPO_4_ (0.1 mmol/L) buffer at pH 6.8, *piggyBac* donor plasmid *M6620* (500 ng/*µ*L), and the *piggyBac* helper plasmid, *phsp‐pBac* (200 ng/*µ*L) (Handler & Harrell Ii, 1999). Microinjection was performed following previously described standard procedures (Handler *et al.*, 1998; Rong & Golic, 2000; Aumann *et al.*, 2018). Briefly, embryos of the *wp* ^−(CRISPR)^ strain were collected for up to 40 min, dechorionized in 1.4% (w/w) solution of sodium hypochlorite for 3 min, rowed on double‐sided sticky tape and covered with halocarbon oil 700 (Sigma Aldrich/Merck KG, Darmstadt, Germany). Needles for injection were made from siliconized quartz glass capillaries (Science Products for Research in Life Science GmbH, Product Number Q100‐70‐7.5, Hofheim, Germany) crafted in a P‐2000 laser puller (Sutter Instruments, Novato, CA, USA). The injection setup consisted of a MN‐151 micromanipulator (Narishige, Tokyo, Japan), a FemtoJet 4i (Eppendorf, Hamburg, Germany), and a SZX16 stereo microscope (Olympus, Tokyo, Japan). After injection, embryos were placed into an oxygen chamber with moistened filter paper at 21 °C. Hatched larvae were carefully transferred into larval food and reared under standard conditions at 25 °C. Eclosed flies were backcrossed to the parental strain, and their progeny were inbred to establish the rescued strain.

### Screening and image acquisition

To identify transformed progeny, pupae were screened for WT color of the puparia and flies, anesthetized with CO_2_, were screened for expression of DsRed fluorescence under a Leica M205FC stereo microscope (DsRed filter: excitation 530–560 nm, emission 590–650 nm). To facilitate image acquisition, flies were cooled down on ice. Pictures were taken using a Leica M205 FCA microscope coupled to a DMC6200 camera and the Leica Application Suite X software (version 3.8.1.26810, Leica, Wetzlar, Germany).

### Nonlethal molecular characterization of *wp* rescue

To molecularly confirm the observed rescued phenotype, nonlethal genotyping was performed according to the Platinum^TM^ Direct PCR Universal Master Mix lysis protocol (Invitrogen, Thermo Fisher Scientific Baltics UAB, Vilnius, Lithuania). Therefore, gDNA was extracted from carefully excised single middle legs of CO_2_‐anesthetized adult flies. Each leg was immediately transferred into 20 *µ*L of lysis solution (containing 0.6 *µ*L of proteinase K to 20 *µ*L of lysis buffer) in a microcentrifuge tube and shortly spun down to ensure complete immersion in the solution. Each fly was kept separately until genotyping was concluded and allowed to recover at standard rearing conditions. The lysis supernatant containing genomic DNA was used for PCR amplification with primers P1634 and P1936, following the manufacturer's instructions. The primers amplify part of the coding sequence from coding exons 1 to 3 of the *white pupae* gene, spanning 135 bp of introns. Therefore, the expected size of the amplicon for the endogenous *wp* gene is 786 bp, while the amplicon from the mini‐*wp* is expected to be 651 bp. The PCR products were analyzed by agarose gel electrophoresis.

### Droplet digital PCR

To confirm the number of integration events in the first generation of positively transformed flies and their heterozygous offspring, droplet digital PCR (ddPCR) was performed using the Auto‐DG System droplet generator from Bio‐Rad, the C1000 Touch Thermal Cycler, and the BIO‐RAD QX200 droplet reader, as previously described (Häcker *et al.*, 2023). Data were analyzed using QuantaSoft Software (Bio‐Rad, Hercules, CA, USA). The mini‐*wp* cassette contained DsRed as the target gene. The primers and dual‐labeled probe used were P49, P50, and DsRed‐probe. Medfly His3 (LOC101459256, encoding histone H3.3) served as the reference housekeeping gene. The primers and dual‐labeled probe included P101, P103, and CCHis3‐probe. DNA restriction digestion prior to droplet generation was performed using EcoRI. Each reaction mix included genomic DNA from heterozygous samples, ddPCR 2× Supermix for probes (without dUTP, Bio‐Rad), a primer‐probe mix, and EcoRI, all reaching a final concentration of 1× Supermix, 900 nmol/L oligonucleotides, 250 nmol/L probes, and 2 U EcoRI in a total volume of 25 *µ*L in a ddPCR 96‐well plate (Bio‐Rad). The cycling conditions for the generated droplets were as follows: 95 °C for 10 min, followed by 40 cycles at 94 °C for 30 sec and 55 °C for 1 min. The enzyme was deactivated at 98 °C for 10 min before the fluorescence of the droplets was analyzed using a droplet reader.

### Inverse PCR and sequence confirmation of integration site

Inverse PCR (iPCR) was performed to determine the genomic location of the *piggyBac* insertions. 600 ng of gDNA, extracted from single virgin flies, was digested with MspI (New England Biolabs Inc., Ipswich, MA, USA) in a 20‐*µ*L reaction at 37 °C for 1 h. Digested DNA was precipitated in 3 mol/L NaOAc and ethanol, and recovered in 50 *µ*L TE buffer. The resuspended DNA was allowed to re‐ligate overnight (approximately 18 h) at 16 °C with 800 Units of T4 DNA ligase (New England Biolabs Inc., Ipswich, MA, USA) in a 350‐*µ*L reaction. Ligated DNA was again precipitated in NaOAc and ethanol, and recovered in 50 *µ*L TE buffer. Inverse PCR reactions were performed using Phusion Flash High‐Fidelity PCR Mastermix and 3 *µ*L of the ligated DNA in a total volume of 20 *µ*L. Primers mfs11 and mfs10 were used to amplify the region flanking the 5' *piggyBac* insertion site, mfs34 and P815 to amplify the region flanking the 3' *piggyBac* insertion site. PCR conditions were as follows: 1× 98 °C 10 s; 5× 98 °C 1 s, 66−56 °C (5' *piggyBac*)/64−54 °C (3' *piggyBac*) (−2 °C per cycle) 5 s, 72 °C 60 s; 35× 98 °C 1 s, 56 °C (5' *piggyBac*)/54 °C (3' *piggyBac*) 5 s, 72 °C 60 s, 1× 72 °C 60 s. PCR products were purified by gel electrophoresis and extracted with the Zymoclean Gel DNA Recovery Kit (Zymo Research Europe GmbH, Freiburg, Germany). Finally, PCR products were Sanger‐sequenced and the results were analyzed for fragments of the *piggyBac* vector and restriction sites of MspI. The sequences were used for BLAST search against the reference genome of *C. capitata* (GCF_000347755.3‐Genome assembly Ccap_2.1) (Papanicolaou *et al.*, 2016) and the assembly version EGII‐3.2.1 (GCA_905071925.1‐Genome assembly EGII‐3.2.1) (Ward *et al.*, 2021) using Geneious Prime. In case of weak or no bands observed in the agarose gel, 1 *µ*L of the iPCR reaction was reserved for a semi‐nested PCR (Shen, 2019). In this case, iPCR product was diluted 1:100 and 1 *µ*L was used for seminested PCR with primers mfs10 and mfs31 for the 5' *piggyBac*, or primers mfs34 and P139 for the 3' *piggyBac* integration site. Cycling conditions were: 1× 98 °C 10 s; 35× 98 °C 1 s, 54 °C (5' *piggyBac*)/52 °C (3' *piggyBac*) 5 s, 72 °C 60 s, 1× 72 °C 3 min. In addition, the integration sites were confirmed by PCR with primers binding in the genomic regions (Table S2) flanking the integration site paired to another primer within the transgene, followed by Sanger sequencing of the produced amplicon. PCR reactions were performed using Phusion Flash High‐Fidelity PCR Mastermix and 40 ng of gDNA in 20‐*µ*L reactions.

### Remobilization of the mini‐*wp piggyBac* cassette

**Fig. 1:**
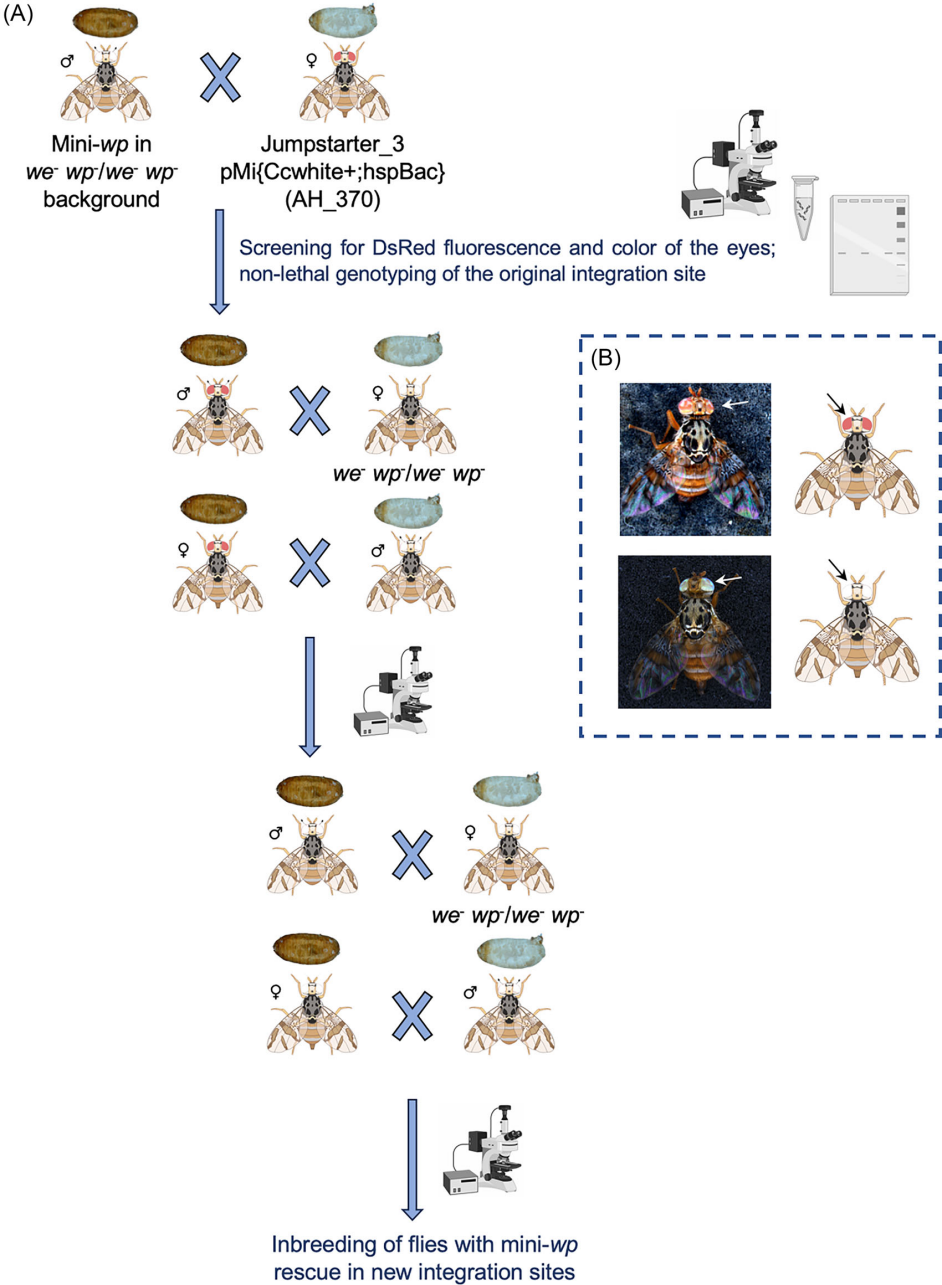
Schematic of remobilization crosses. (A) Crosses for remobilizing the *piggyBac* integrated mini‐*wp* rescue cassette using the Jumpstarter 3 strain as the source of transposase. The color of the eyes, indicating the presence (peach‐colored eyes, top) or absence (white eyes, bottom) of the Jumpstarter cassette, is highlighted in B and also displayed in the schematic of the flies in A. Partially created with BioRender.com.

To generate strains with new genomic integration sites of the mini‐*wp piggyBac* construct, we used the *piggyBac*‐Jumpstarter 3 strain (Schetelig *et al.*, 2009) as a transposase source. This strain contains the pMi{Ccwhite+; hspBac} cassette (AH_370) (Schetelig *et al.*, 2009), which expresses a *piggyBac transposase* and the medfly *white* gene. This setup allows for the remobilization of *piggyBac* constructs like the mini‐*wp* and the rescue of the white eye phenotype. Consequently, eye color can be used as a marker to confirm the presence of the Jumpstarter cassette after remobilization, particularly when using a strain with a white eye phenotype in the experiments. Therefore, it was necessary to first obtain the mini‐*wp* rescue within a *white eye/white pupae* background: The mini‐*wp* strain with the original integration site was outcrossed to a strain double homozygous for natural mutations of the *white eye* and *white pupae* (*we* ^−^ *wp* ^−^/*we* ^−^ *wp* ^−^). After establishing a mini‐*wp* rescued strain with *we* ^−^ *wp* ^−^/*we* ^−^ *wp* ^−^ background, males and females of this strain were mated with their counterparts of the Jumpstarter 3 strain. The offspring were screened for DsRed fluorescence, the color of the eyes, and nonlethally genotyped with primers binding to the genomic DNA flanking the original integration site and primers inside the mini‐*wp* cassette to determine whether the cassette remained in the original integration site or possibly remobilized. Primers P2357, P2358, and P2273 were used on the 5' *piggyBac* end, while mfs34 and P2359 were used on the 3' *piggyBac* end. To facilitate identification of the flies during genotyping and crosses, all flies were consecutively numbered and the families established thereof were named after their numbers. The family established from the fly with the original integration site was named after the plasmid number (M6620). Flies with potentially new integration sites were individually outcrossed with counterparts of the strain *we* ^−^ *wp* ^−^/*we* ^−^ *wp* ^−^. The offspring was again screened and rescued flies with white eye phenotype were individually outcrossed with the *we* ^−^ *wp* ^−^/*we* ^−^ *wp* ^−^ strain. The white eye phenotype indicates the absence of the Jumpstarter cassette, therefore, indicating that the new integration sites have been successfully stabilized. The offspring was then screened again and rescued flies were inbred to establish strains of mini‐*wp* rescued flies in the *we* ^−^ *wp* ^−^/*we* ^−^ *wp* ^−^ background with different integration sites of the *piggyBac* cassette (Fig. 1). Integration sites of the new strains were identified through iPCR and compared to the available genomic sequences of the medfly, as described above. Flanking sequences of the integration sites for all mini‐*wp* strains are given in Table S2.

### Mini‐*wp* functional rescue capability from different genomic positions

To verify the functionality of the mini‐*wp* construct to rescue the *white pupae* phenotype when integrated in different genomic positions, homozygous flies expressing the mini‐*wp* rescue were outcrossed to their homozygous counterparts of a strain carrying the natural mutation of the *white pupae* gene. Eggs were collected on two consecutive days between oviposition days 5 to 8, in two to six batches, depending on the total number of laid eggs. Ideally, 100 eggs were collected from each cage per batch. After collection, each batch of eggs was reared separately. Pupae were screened for DsRed fluorescence and puparium color and counted as “rescued” (brown puparium) or “white pupae” (white puparium). Heterozygous adults eclosing from rescued pupae were screened for DsRed fluorescence and again outcrossed to their homozygous counterparts of a strain with the natural mutation of the *wp* gene. Again, pupae were screened and counted as rescued or white pupae. Rescue capability is given as percentage of pupae with rescued phenotype in relation to the total amount of pupae obtained. Numbers of collected eggs, pupae and adults for each experiment are given in Tables S3–S5.

### Statistical analysis

Data analysis was carried out using the software MiniTab^®^ (Minitab, LLC., State College, CA, USA). Data were analyzed via one‐way analysis of variance (ANOVA), with Ryan–Joiner test for normality and Levene's test for equal variance. The means were then compared with the Tukey's simultaneous test for differences of means at 95% confidence level. All statistical analyses are provided in Table S6.

## Results

### Analysis of the *white pupae* gene structure and cloning of a minimal gene fragment into a *piggyBac* vector

**Fig. 2:**
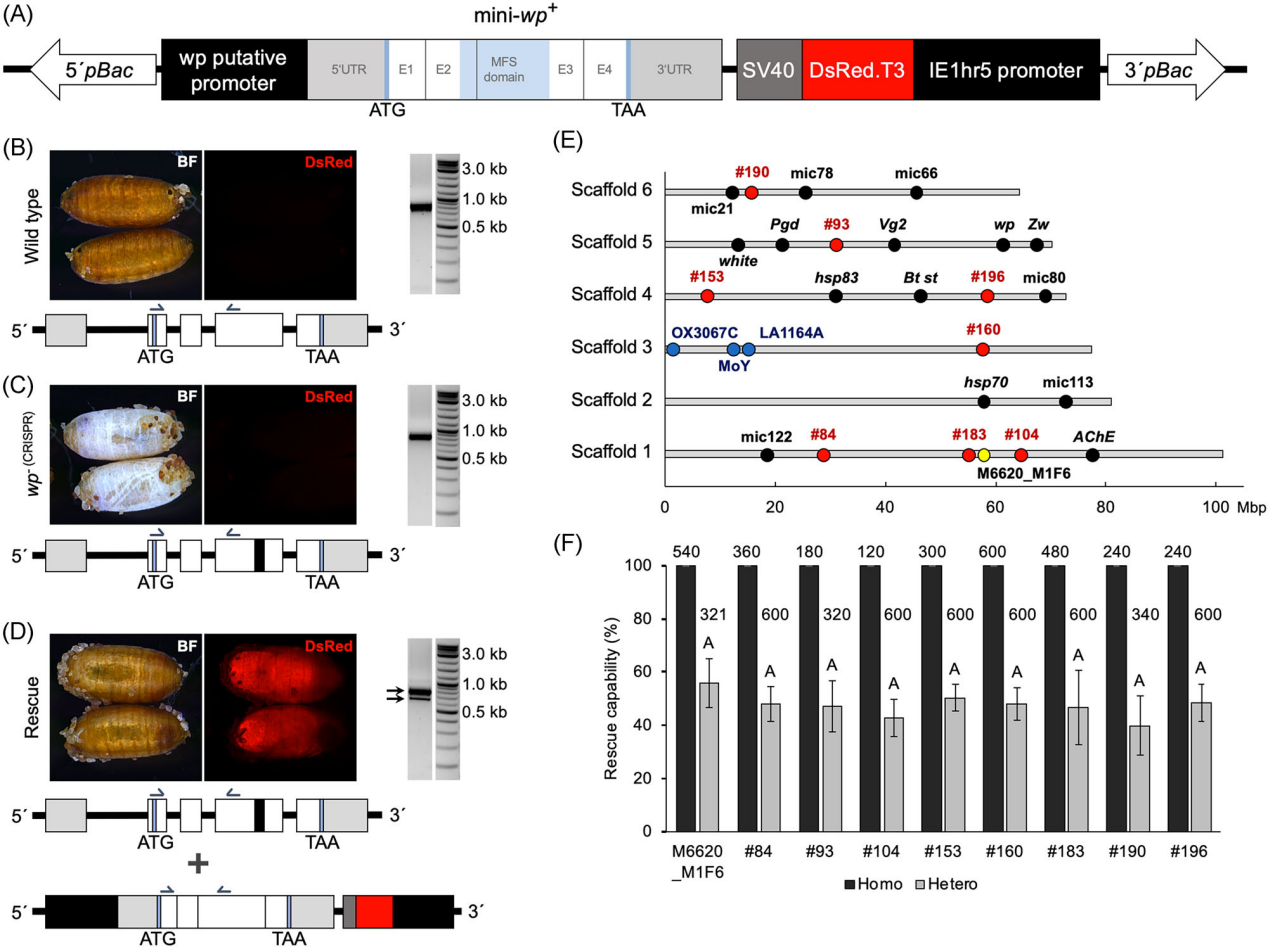
Restoring the wild‐type brown pupae phenotype via *white pupae* gene integration. (A) Schematic of the mini‐*white‐pupae* construct in a *piggyBac* vector. The total size of the *piggyBac* cargo is 6856 bp. ATG and TAA represent the position of the *wp* start and stop codons, respectively. E1, E2, E3, E4 represent the coding exon count. The Major Facilitator‐like superfamily (MFS) domain is indicated in light blue. Phenotype of the wild‐type allele of the *white pupae* gene (EgII strain) is shown in B, compared to a *wp* ^−(CRISPR)^ strain (C), and the phenotype observed after germline transformation of the mini‐*wp* gene, that is, the phenotype of the rescued strain in D. A schematic of their respective *wp* gene version(s) is shown below each phenotype figure. Semiarrows in the schematic of the genes represent primer binding sites used to molecularly differentiate between the different *wp* alleles. Black stripe in the third coding exon of the *wp* gene in C and D represents the CRISPR‐mediated deletion reported to cause the white pupae phenotype (Ward *et al.*, 2021). 1 kb plus ladder (New England Biolabs Inc., Ipswich, MA, USA) was used as marker in gel electrophoresis. kb, kilobases; BF, bright field; DsRed, fluorescence filter. (E) The integration sites mapped to the medfly genome assembly version 3.2.1 (GCA_905071925.1). Yellow dot indicates the original *piggyBac* integration site obtained through germline transformation; red dots indicate integration sites following remobilization of the original integration site; black dots denote reference genes, known genomic regions, or microsatellite (*mic*) sequences previously mapped to autosomal chromosomes or the Y chromosome (blue dots) (Condon *et al.*, 2007; Papanicolaou *et al.*, 2016; Meccariello *et al.*, 2021; Ward *et al.*, 2021). *AChE–Acetylcholinesterase*, *hsp70–heat shock 70*, *MoY–Maleness‐on‐the‐Y*, *hsp83–partial gene for heat shock protein 83*, *Bt st–Bactrocera tryoni scarlet gene*, *Pgd–6‐phosphogluconate dehydrogenase*, *Vg2–Vitellogenin 2*, *wp–white pupae*, *Zw–glucose‐6‐phosphate 1 dehydrogenase*; OX3067C and LA1164A are Y‐specific *piggyBac* transposon 5'‐flanking sequence from transgenic lines reported in the literature (Condon *et al.*, 2007). (F) Rescue capability of mini‐*wp* integrated in different genomic positions. Shown are the mean values in percentage of offspring that expressed the restored WT phenotype at pupal stage (rescue capability). Data shown are based on 2– 6 batches of egg collections. Total number of eggs collected are given above the bars. Error bars indicate the standard deviation and bars with common letter are not significantly different at 95% confidence level (one‐way ANOVA).

The structure of the *white pupae* gene (LOC101451947) (Ward *et al.*, 2021), *in silico* annotated with a total length of 20868 bp and coding sequence (CDS) of 1596 bp over four coding exons, was successfully verified via Rapid Amplification of cDNA Ends (RACE) PCR. Mapping the obtained sequences to the medfly reference genome (GCF_000347755.3) (Papanicolaou *et al.*, 2016) placed the start of the 5' UTR at 18489 bp upstream the start of the predicted CDS, signaled by the starting codon, as suggested in the annotated mRNA version XM_004530458.4. On the other hand, the 3' UTR was mapped to the version XM_020860083.1. As shorter cargo sequences might facilitate insect transgenesis, a minimal gene version of the *white pupae* gene (mini‐*wp*, m*wp*) was designed to help subsequent insect transgenesis efforts. A putative endogenous promoter region (2000 bp), along with the 5' UTR, the CDS and the 3' UTR of the *white pupae* gene, in total 4689 bp, were cloned into a *piggyBac* vector containing a DsRed fluorescent marker (Fig. 2A).

### Germline transformation with mini‐*wp* restores wild‐type pupal phenotype

Germline transformation was performed through microinjection into 849 embryos of a *wp* ^−(CRISPR)^ strain (Ward *et al.*, 2021). All G_0_ adults, eight males and eight females, were individually backcrossed to the parental *wp* ^−(CRISPR)^ strain. Offspring (G_1_) were screened for DsRed fluorescence and color of the puparium at the pupal stage and one out of 124 pupae from the female #6 showed the rescued phenotype, that is, wild‐type brown puparium color and expression of DsRed fluorescence. The transgene copy number in the rescued individual and its heterozygous progeny was confirmed to be a single copy by ddPCR, indicating a unique *piggyBac* integration event. The rescued male fly (M6620_M1F6) was backcrossed to females of the *wp* ^−(CRISPR)^ strain and their progeny were inbred to establish the rescued strain (Figs. 2D and S2). PCR amplification using primers that span the two introns between coding exons 1 and 3 of the *wp* gene, performed on gDNA, made it possible to molecularly distinguish whether the brown puparium color was caused by the engineered mini‐*wp* or the wild‐type (WT) allele of the *wp* gene (Fig. 2B−D).

### Transposase‐mediated remobilization integrates mini‐*wp* in different genomic positions

To verify that the mini‐*wp* rescue construct could be integrated and functional in other genomic positions within the medfly genome, the Jumpstarter 3 strain (Schetelig *et al.*, 2009) was used as source of *piggyBac* transposase to remobilize the mini*‐wp* cassette from its original integration site. Following the crossing scheme depicted in Fig. 1, eight new strains with the rescued phenotype have been established. The integration sites for all rescued strains were determined via iPCR, and the genomic region flanking the integration site confirmed by Sanger sequencing (Table S2). Notably, integration site of strain #160 was identified in a scaffold region predicted to be the chromosome X. The integration sites of all other strains were identified in autosomal positions, and based on *in silico* analysis of annotated genes and microsatellite sequences (Papanicolaou *et al.*, 2016), likely to be on chromosomes 2 (scaffold 1), 4 (scaffold 6), 5 (scaffold 5), and 6 (scaffold 4) (Fig. 2E).

### Mini‐*wp* successfully rescues WT pupal phenotype from different integration sites

To functionally evaluate the mini‐*wp* rescue integrated in different genomic positions, flies from the rescued strains were crossed to flies carrying the natural mutation of the *white pupae* gene, reported as an insertion of ∼8150 bp in the third coding exon (Ward *et al.*, 2021). The offspring was screened for the rescued WT phenotype and DsRed fluorescence at pupal stage and adults were outcrossed again to evaluate the rescue capability in heterozygosity. The rescue from all evaluated positions was fully functional, with 100% of the offspring from homozygous crosses showing the restored WT phenotype at pupal stage. Additionally, the rescue was also functional in heterozygosity, that is, a single copy of the mini‐*wp* gene was sufficient to rescue a homozygous *wp^−^* mutant (Fig. S3). Consistent with Mendelian inheritance patterns, ∼50% of the offspring expressed the rescued pupal phenotype when crossing m*wp* ^±(nat)^ flies with *wp* ^−(nat)/‐(nat)^ mutants (47.43% ± 4.5%, averaging across all strains with different integration sites of the mini‐*wp*, with no significant statistical difference between the strains (*P*‐value = 0.297, one‐way ANOVA). To functionally validate the integration predicted to be on the X chromosome, we outcrossed males of strain #160 to their counterparts carrying the natural mutation on the *wp* gene. As expected for a marker located on the X chromosome, all rescued offspring were female, while all flies that eclosed from the white puparia were males (Table 1). Interestingly, in a subsequent round of rearing for maintenance of the line #160, when outcrossing the rescued male offspring to females of the *wp^‐^* strain, we observed two males that eclosed from rescued pupae. In this occasion, a total of 600 eggs were collected. Upon the hypothesis that a recombination event might have occurred, these males were outcrossed to females of the *wp^−^* strain. Despite several egg collections, no adult offspring was obtained and it was not possible to establish new families from these male flies. As these males did not survive or produce offspring, no further analysis was carried out as part of this work. Lines #104 and #183 were not viable after approximately five generations of inbreeding, suggesting that the integration of the rescue cassette in the different lines might have fitness costs, impacting their maintenance.

**Table 1:** Inheritance distribution of rescued phenotype in the offspring of male medflies with the mini‐*wp* integrated into the X chromosome crossed to homozygous *wp^−^* females

Phenotype	Number of pupae	Number of eclosed males	Number of eclosed females
Rescued	208	0	200
White pupae	263	247	0

### Unexpected phenotypes derived from remobilization of the piggyBac integration

**Fig. 3:**
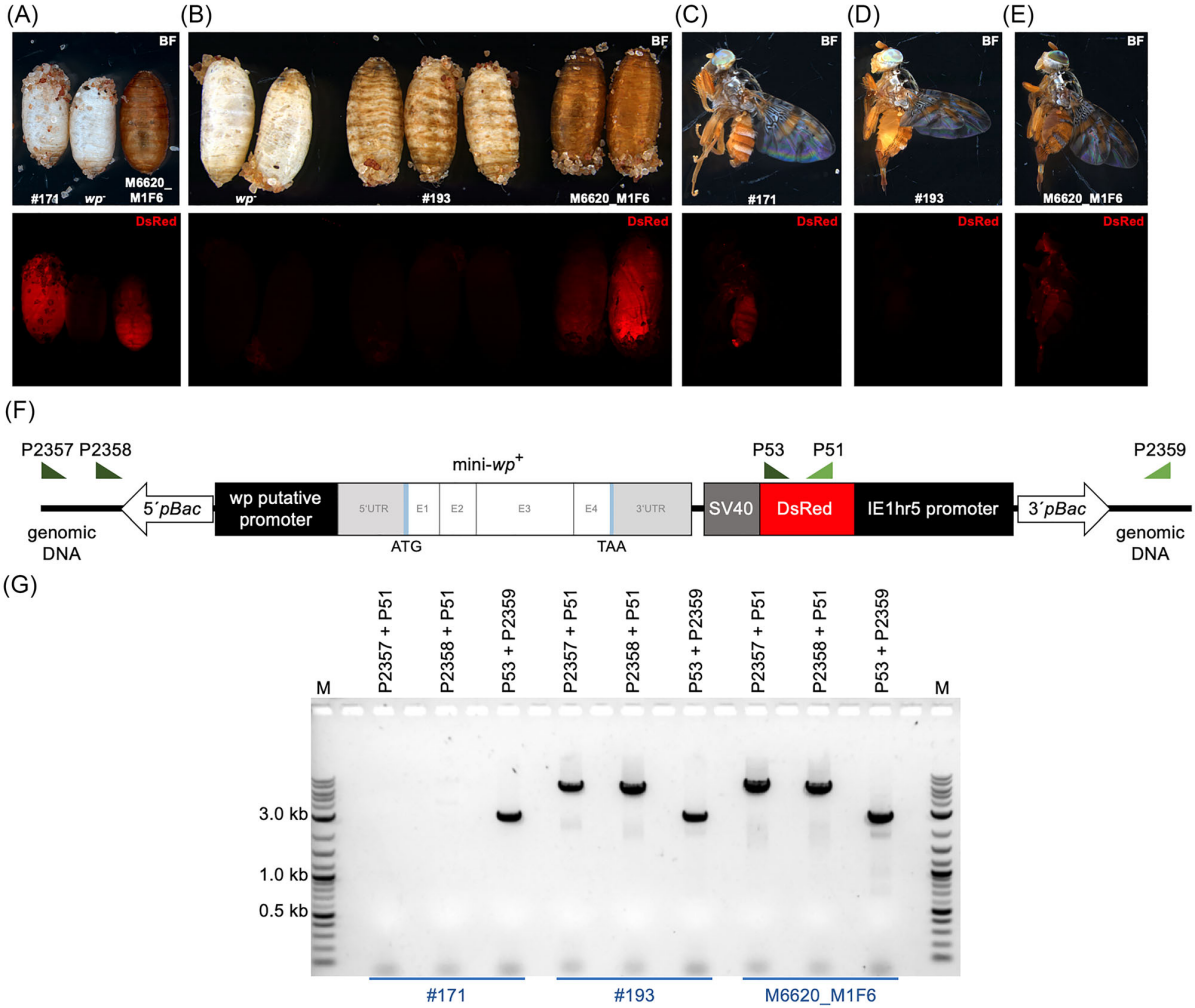
Unexpected phenotypes obtained from remobilization of the mini‐*wp*‐DsRed *piggyBac* cassette. (A) Phenotypes of strain #171 at pupal stage (left), natural mutated *white pupae* strain (middle), and rescued strain M6620_M1F6 (right). (B) Two pupae from the natural mutated *white pupae* strain (left), three pupae with segmented patterns of WT puparium color (#193) (middle), two pupae from the rescued strain M6620_M1F6 (right). Adults from strains #171 (C), #193 (D), and M6620_M1F6 (E). Position of forward (P2357, P2358, P53) and reverse (P51, P2359) primers used to check integration of the rescue cassette are schematically shown in F. The original integration site was analyzed using genomic DNA of all three strains with the following primer combinations: P2357 and P51 (expected amplicon size: 6455 bp), P2358 and P51 (expected amplicon size: 6080 bp), P53 and P2359 (expected amplicon size: 2839 bp). PCR products analyzed via gel electrophoresis are shown in G. 1 kb plus ladder (New England Biolabs Inc., Ipswich, MA, USA) was used as marker (M) in gel electrophoresis. kb, kilobases; BF, bright field; DsRed, fluorescence filter.

Among the flies obtained from the remobilization experiment, flies #171 and #193 stood out with unexpected phenotypes. Fly #171 showed no rescued phenotype of the puparium color, but positive expression of the fluorescent marker (Fig. 3A, C). PCR amplification flanking the genomic position of the original integration site showed partial amplification of the cassette on the 3' end of the integrated cassette in the original position. However, amplification spanning the 5' end of the cassette was not possible, suggesting that part of the construct is missing (Fig. 3G). It was also not possible to amplify parts of the mini‐*wp* cassette upstream from the fluorescent marker (Fig. S4), suggesting that part of the cassette might have been excised out. Fly #193 presented a segmented WT phenotype of the puparium (Fig. 3B), and no expression of the fluorescent marker (Fig. 3B, D). PCR amplification on both 3' and 5' ends of the rescue cassette confirmed the integration site of the original strain (M6620_M1F6), suggesting that there was no remobilization (Fig. 3G).

## Discussion

The concept of neo‐classical GSS provides a generic approach for engineering GSS across various insect pest species in a targeted and time‐efficient manner (Häcker *et al.*, 2021; Nguyen *et al.*, 2021; Yan *et al.*, 2023; Yan *et al.*, 2024). Unlike classical GSS, which depend on random or induced mutations, the neo‐classical approach employs genome editing for greater precision and efficiency (Ward *et al.*, 2021; Chen *et al.*, 2022; Sollazzo *et al.*, 2024). CRISPR/Cas can induce targeted mutations in marker genes, facilitating phenotypic mutant strains. Additionally, this approach may overcome issues of semisterility and recombination associated with irradiation‐induced translocations by precisely integrating the WT allele into the Y chromosome or near a male‐determining factor (Franz *et al.*, 2021; Cáceres *et al.*, 2023).

Here, we investigated the *white pupae* gene as a potential marker for the generic approach to construct neo‐classical GSS and demonstrated that the wild‐type brown pupal color phenotype can be restored through *piggyBac*‐mediated insertion of an intronless version of the *wp* gene (mini‐*wp)* into the genome of a *white pupae* mutant strain.

Previous studies indicate that HDR‐mediated knock‐in efficiency decreases as cargo size increases (Li *et al.*, 2014; Paix *et al.*, 2017), and that targeted insertions may be particularly challenging in repetitive and heterochromatic regions, such as the Y chromosome (Bernardini *et al.*, 2014; Buchman & Akbari, 2019). Consequently, using the complete *white pupae* gene to establish sex‐linkage in neo‐classical GSS might present a challenge, as the cargo would reach approximately 20 kb due to an 18 kb intron located between the 5' UTR and the start codon of the *wp* gene. To address this challenge, we designed a minimal, intronless version of the *wp* gene with only 4689 bp, including a 2000 bp genomic sequence upstream of the 5' UTR that we used as the putative promoter region. The successful *piggyBac*‐mediated integration of the mini‐*wp* and the resulting restoration of the WT pupal phenotype in a *wp* ^−(CRISPR)^ strain demonstrates the feasibility of obtaining complete phenotypical rescue using an intronless *wp* gene. Although not investigated in this study, it may be possible to further reduce the size of the mini‐gene by condensing the CDS to include only its functional domains or by using a shorter endogenous promoter region. The latter possibility is supported by recent findings where a minimal version of the *Z. cucurbitae wp* gene remained functional after the promoter region was reduced from 2000 bp to 605 bp (Fan *et al.*, 2025).

A PCR assay using primers spanning the introns of the *wp* gene enables differentiation between brown pupae phenotype flies carrying the endogenous WT alleles (*wp^+^/wp^+^*) and those with at least one copy of the engineered mini‐*wp* allele in a *white pupae* mutant background (*wp* ^‐^/m*wp* ^‐^) (Fig. 2B, D). This approach is valuable for quality control in mass‐rearing facilities and for identifying recaptured flies in SIT programs. Furthermore, it eliminates the need for a linked fluorescent marker, allowing the rescue construct to consist entirely of endogenous sequences, which may facilitate regulatory approval. If regulatory frameworks exempt targeted modifications that introduce only naturally occurring sequences—even with minor changes such as small deletions—from GMO classification, then insects engineered with such minimal modifications could be considered equivalent to conventionally bred insects and thus not subject to GMO regulations. This could have significant implications for regulatory acceptance and public perception.

During the maintenance of strains generated in the remobilization experiment, we observed variations in overall performance (Table S3), with two out of nine strains (#104 and #183) unable to be maintained. This variability likely stems from the random nature of *piggyBac* integration, as insertions into essential genes or regulatory regions can disrupt gene expression and impair fitness. In strain #104, for example, the m*wp* integration site was located within a predicted protein‐coding gene, cyclic nucleotide‐binding domain‐containing protein 2, which may have affected viability. Nonetheless, the other seven strains have been maintained under laboratory conditions and stably expressed the restored WT phenotype for over ten generations. In strain #171, the white pupal phenotype accompanied by *DsRed* expression (Fig. 3A, G) suggests partial excision of the m*wp* cassette, leaving only the fluorescent marker and the 3’ end of the *piggyBac* vector at the original site. Similarly, the WT‐striped phenotype observed in strain #193 may result from mutations affecting the regulatory region of *wp* or other genetic elements. However, these mutations do not explain the absence of fluorescent marker expression. The link between this phenotype and the remobilization experiment remains unclear, and further investigation was beyond the scope of this study.

For advancing the engineering of neo‐classical GSS, CRISPR/Cas technology could be employed to integrate mini*‐wp* precisely into specific regions of the Y chromosome in *white pupae* phenotypic strains, ensuring stable and sex‐specific expression. Achieving high‐quality sequencing of the Y chromosome and establishing reliable protocols for precise gene editing of the Y are the next challenges, along with identifying optimal target sites on the Y chromosome to ensure robust expression and stable integration of the construct. The solid foundation of gene function and gene editing research in *C. capitata* (Meccariello *et al.*, 2017; Aumann *et al.*, 2018; Meccariello *et al.*, 2019; Aumann *et al.*, 2020; Meccariello *et al.*, 2021; Ward *et al.*, 2021; Sollazzo *et al.*, 2024) underscores the importance of this species for proof‐of‐concept experiments and for the further development of neo‐classical GSS. Moreover, the well‐established classical GSS, VIENNA‐7 and VIENNA‐8, combined with extensive experience in mass rearing, will likely facilitate future large‐scale experiments, including assessments of fitness in these engineered strains. The toolkit developed for designing and validating the intronless *wp* gene could be extended to related species where this gene has been identified and *wp* ^‐(CRISPR)^ strains have already been generated (Ward *et al.*, 2021; Paulo *et al.*, 2022). Expanding this approach across multiple SIT‐target species would enhance the versatility and applicability of neo‐classical GSS.

Overall, our findings represent a significant step toward developing neo‐classical GSS for SIT applications. We have demonstrated that the WT phenotype can be restored by integrating an intronless *wp* gene into various genomic sites in *Ceratitis capitata*. This study provides a foundation for future gene‐editing efforts to engineer GSS in other SIT‐target species, leveraging modern molecular tools to develop robust and efficient sex‐specific selectable markers. Further refinements, including optimizing integration sites and assessing long‐term stability, will be crucial for advancing this approach toward practical implementation.

## Author contributions

Conceptualization: LHFP, MFS, RAA; Data curation: LHFP; Formal analysis: LHFP; Funding acquisition: LHFP, MFS; Investigation: LHFP, IS, TR; Methodology: LHFP, MFS, RAA; Project administration: LHFP, MFS; Resources: MFS; Supervision: MFS, RAA; Visualization: LHFP; Writing—original draft: LHFP; Writing—review & editing: LHFP, MFS, RAA.

## Disclosure

The authors declare no conflicts of interest. The funders had no role in the design of the study; in the collection, analyses, or interpretation of data; in the writing of the manuscript; or in the decision to publish the results.

## Supporting information




**Fig. S1** Schematic on the endogenous *white pupae* and minimal gene construct.
**Fig. S2** Schematic of crosses for establishment of mini‐*wp* strain.
**Fig. S3** Schematic of crosses for functional evaluation of mini‐*wp* (m*wp* ^+^) strain.
**Fig. S4** PCR amplification of different parts within the mini‐*wp* rescue in strains #171 and #193.

**Table S1** Primer sequences used in this study.
**Table S2** Flanking sequences of the integration site for all mini‐*wp* strains.
**Table S3** Rescue capability in homozygosity.
**Table S4** Rescue capability in heterozygosity.
**Table S5** Rescue of strain #160 with mini‐*wp* on the X chromosome.
**Table S6** Statistical analysis.

## Data Availability

Mini‐*wp* strains and vectors (*AH465* and *M6620*) can be requested from MFS.
