# Supplementary material for: Functional validation of a white pupae minimal gene construct in Ceratitis capitata (Diptera: Tephritidae)
Source: Insect Sci. 2025 May 12;33(2):491–504. doi: 10.1111/1744-7917.70058 (PMC13087832; doi:10.1111/1744-7917.70058)
Supplement: Supplementary file 1 — Fig. S1 Schematic on the endogenous white pupae and minimal gene construct. Fig. S2 Schematic of crosses for establishment of mini‐wp strain. Fig. S3 Schematic of crosses for functional evaluation of mini‐wp (mwp +) strain. Fig. S4 PCR amplification of different parts within the mini‐wp rescue in strains #171 and #193. [file INS-33-491-s002.pdf]

Functional validation of a *white pupae* minimal gene construct in *Ceratitis capitata* (Diptera: Tephritidae)

Lucas Henrique Figueiredo Prates, Roswitha A. Aumann, Inga Sievers, Tanja Rehling, Marc F. Schetelig*

*Correspondence: Marc.Schetelig@agrar.uni-giessen.de

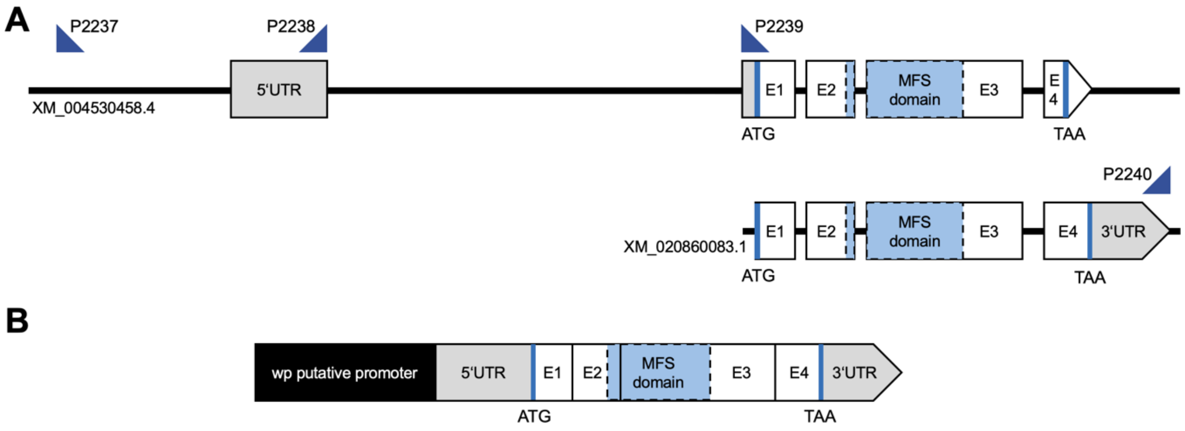

Figure S1. **Schematic on the endogenous *Ceratitis capitata white pupae* and minimal gene construct.** Schematic of the annotated mRNA versions XM_004530458.4 and XM_020860083.1 are shown in **(A)**. The construction of the minimal gene construct **(B)** was done using primers P2237 and P2238 on genomic DNA (gDNA) and primers P2239 and P2240 on cDNA. E1, E2, E3, E4 represent the coding exon count. The Major Facilitator-like superfamily (MFS) domain is indicated in light blue.

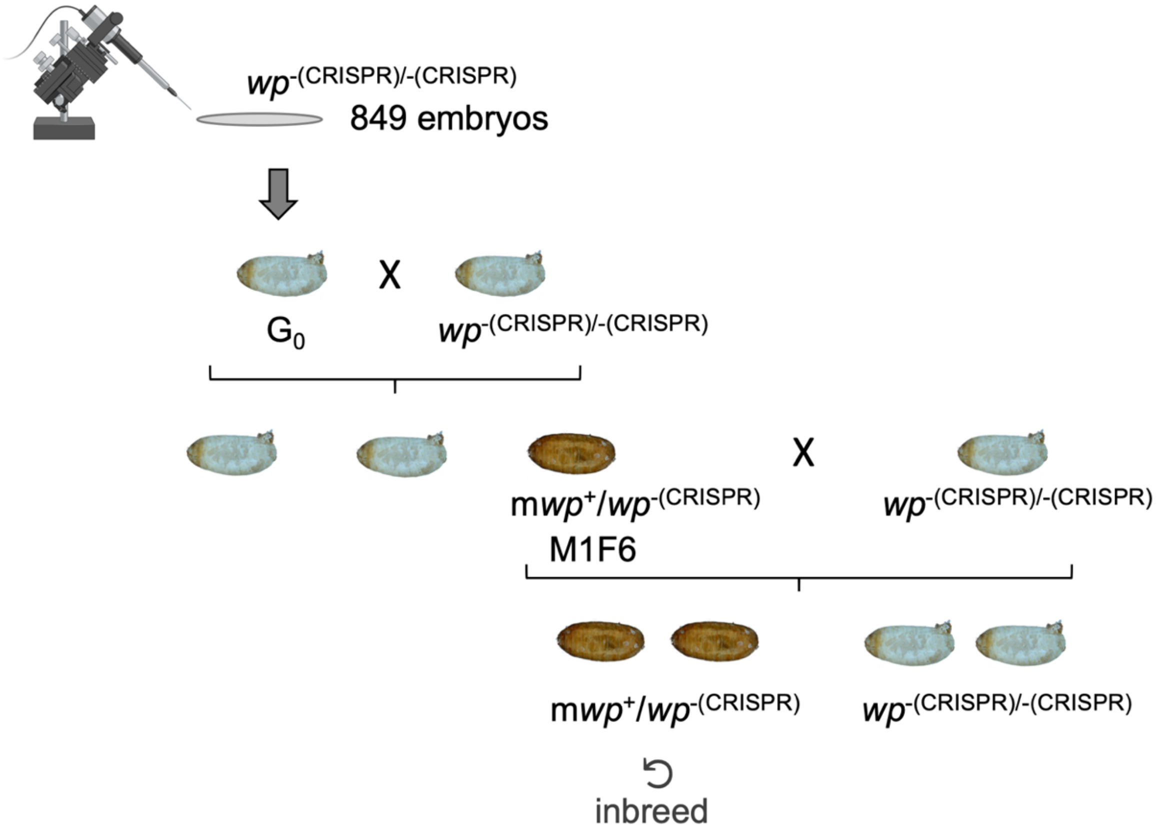

Figure S2: Schematic of the crosses used to establish the mini-*wp* (m*wp*$^+$) strain. Following germline transformation via microinjection into *wp*$^{-(\mathrm{CRISPR})}$ strain embryos, G_0 individuals were individually backcrossed to the parental strain. Offspring exhibiting the rescued phenotype, i.e., wild-type brown puparium color and DsRed fluorescence (fluorescence not shown in this schematic), were backcrossed to the *wp*$^{-(\mathrm{CRISPR})}$ strain. The rescued progeny were then inbred to establish the homozygous mini-*wp* (m*wp*$^+$) strain.

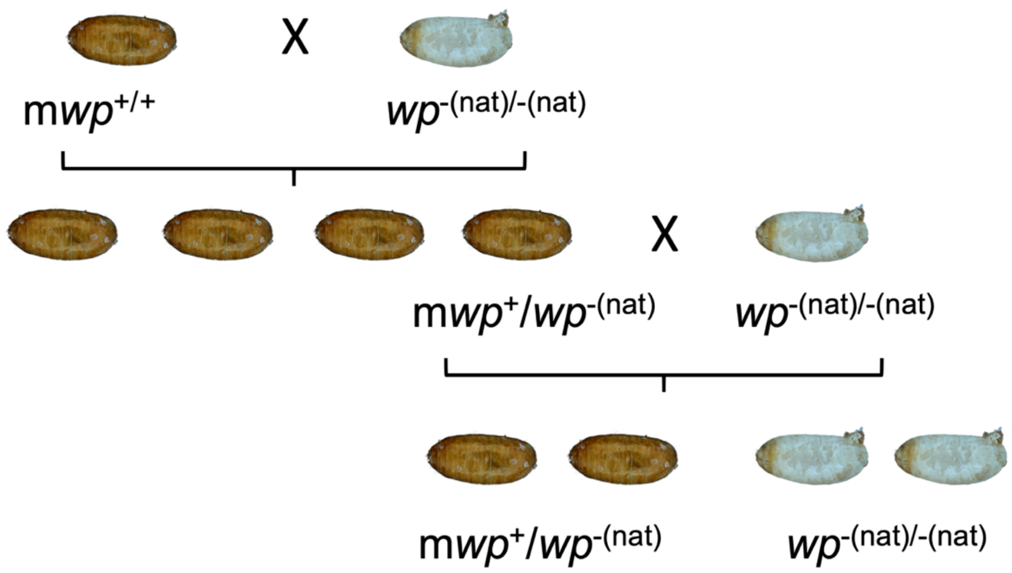

Figure S3: Schematic of the crosses used for the functional evaluation of the mini-*wp* (m*wp*$^+$) strain. Homozygous mini-*wp* (m*wp*$^{+/+}$) flies were crossed with flies carrying the natural mutation of the *white pupae* gene (*wp*$^{-(nat)/-(nat)}$). Offspring were screened for the restored WT phenotype and DsRed fluorescence at the pupal stage. Adults were then outcrossed to the *wp*$^{-(nat)/-(nat)}$ strain to assess the rescue capability in heterozygotes. DsRed fluorescence is not depicted in this schematic.

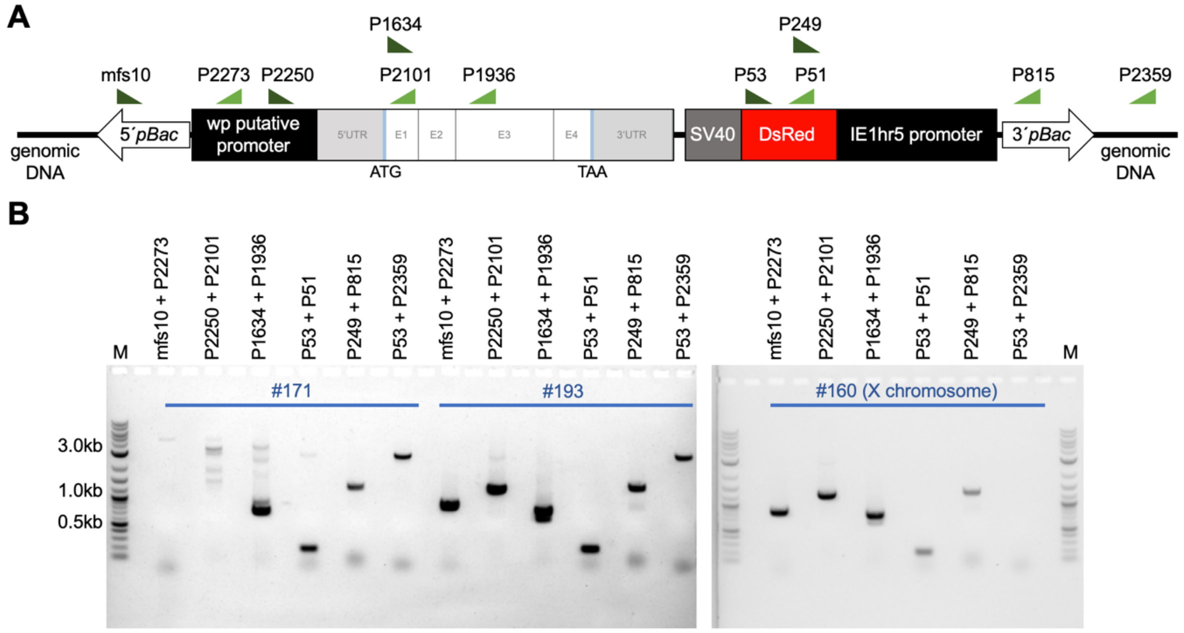

C

Primer combination	Expected amplicon size
mfs10 + P2273	883 bp
P2250 + P2101	1286 bp
P1634 + P1936	649 bp
P53 + P51	200 bp
P249 + P815	1348 bp
P2359 + P53	2839 bp

Figure S4. **Molecular analysis of the unexpected phenotypes obtained from remobilization of the mini-*wp*-DsRed *piggyBac* cassette.** (**A**) Position of primers used to amplify different parts within the *mwp* rescue cassette are schematically shown. (**B**) PCR products amplified from genomic DNA of strains #171, #193, and #160 were analyzed via gel electrophoresis. Primer combinations and expected sizes of the amplicons are given in (**C**). P2359 is specific to the genomic position in the original integration site (M6620_M1F6), thus amplification with gDNA from strain #160 is not expected. M: Marker, 1 kb plus ladder (New England Biolabs Inc., Ipswich, MA, USA); kb = kilobases. E1, E2, E3, E4 represent the coding exon count.
